# A structure- and chemical genomics-based approach for repositioning of drugs against VCP/p97 ATPase

**DOI:** 10.1038/srep44912

**Published:** 2017-03-21

**Authors:** Aldo Segura-Cabrera, Reshmi Tripathi, Xiaoyi Zhang, Lin Gui, Tsui-Fen Chou, Kakajan Komurov

**Affiliations:** 1 Red de Estudios Moleculares Avanzados, Instituto de Ecología A.C., Xalapa, Veracruz, México; 2 Division of Experimental Hematology and Cancer Biology, Cincinnati Children’s Hospital Medical Center, OH, USA; 3 Division of Medical Genetics, Department of Pediatrics, Harbor–UCLA Medical Center and Los Angeles Biomedical Research Institute, Torrance, CA, USA

## Abstract

Valosin-containing protein (VCP/p97) ATPase (a.k.a. Cdc48) is a key member of the ER-associated protein degradation (ERAD) pathway. ERAD and VCP/p97 have been implicated in a multitude of human diseases, such as neurodegenerative diseases and cancer. Inhibition of VCP/p97 induces proteotoxic ER stress and cell death in cancer cells, making it an attractive target for cancer treatment. However, no drugs exist against this protein in the market. Repositioning of drugs towards new indications is an attractive alternative to the *de novo* drug development due to the potential for significantly shorter time to clinical translation. Here, we employed an integrative strategy for the repositioning of drugs as novel inhibitors of the VCP/p97 ATPase. We integrated structure-based virtual screening with the chemical genomics analysis of drug molecular signatures, and identified several candidate inhibitors of VCP/p97 ATPase. Importantly, experimental validation with cell-based and *in vitro* ATPase assays confirmed three (ebastine, astemizole and clotrimazole) out of seven tested candidates (~40% true hit rate) as direct inhibitors of VCP/p97 and ERAD. This study introduces an effective integrative strategy for drug repositioning, and identified new drugs against the VCP/p97/ERAD pathway in human diseases.

The endoplasmic reticulum (ER) is the site of convergence of multiple signaling and metabolic pathways to regulate protein homeostasis (proteostasis)^1^. The ER proteostasis network involves multiple interconnected pathways for balancing the protein folding capacity of the ER with its client protein load. Newly synthesized membrane and extracellular proteins are imported into the ER, where they are rapidly N-glycosylated and folded by the ER N-glycosylation and folding machinery. While folded proteins are exported to Golgi for further processing, misfolded proteins are either refolded, or cleared from the ER by the action of the ER-associated degradation (ERAD) pathway^1^.

ERAD is an essential component of the ER protein homeostasis, as it promotes the “clearance” of excess misfolded proteins from the ER by transporting them across the ER to the cytoplasm and targeting them for proteasomal degradation^2^. ERAD is a multi-component system that involves the recognition of misfolded proteins, their transport through the ER membrane into the cytoplasm and the delivery to the proteasome for degradation. The VCP/p97 ATPase has been found to be an essential member of the ER translocation and proteasomal delivery functions of ERAD, as its inhibition impaired ERAD and caused proteotoxic stress characterized by the accumulation of poly-ubiquitinated protein aggregates. As such, VCP/p97 is an attractive target in diseases involving excessive ERAD, such as cystic fibrosis and cancer.

VCP/p97 is a AAA+ ATPase that assembles as a hexameric double ring machine formed by six identical monomers. Each monomer is composed of three domains; the N-terminal, D1 ATPase and D2 ATPase domains^3^,^4^,^5^. Several potent and specific VCP/p97 inhibitors have been developed, including those that act in an allosteric (NMS-873^6^ and UPCDC30245^7^) or competitive manner (DBeQ^8^, ML240^9^, ML241^9^ and CB-5083^10^). However, although one of these drugs (CB-5083) is currently in the early-phase clinical trials, there are currently no drugs in the market that target the VCP/p97 or any of the ERAD components, and the fate of CB-5083 in the clinical trials is unknown.

The drug development process is a daunting task that consumes huge amounts of resources. Drug repositioning has emerged as an increasingly popular approach to speed up the drug discovery process by finding new uses for approved drugs, thereby significantly reducing the cost and time of drug development^11^. For example, thalidomide, which was withdrawn for its deleterious effects on fetal development, has re-emerged as a drug of great interest for leprosy and multiple myeloma treatment because of its beneficial immunomodulatory effects^12^,^13^,^14^,^15^,^16^,^17^. However, the successes in drug repositioning have primarily been by serendipitous discovery or clinical observation, such as the new indications for thalidomide^18^,^19^. Several chemoinformatics, bioinformatics and network-based strategies have been developed to transform the serendipitous process into a rational and exhaustive drug repositioning approach^20^,^21^,^22^,^23^,^24^,^25^,^26^,^27^,^28^,^29^. Here, we have conducted an integrative approach, where structure-based virtual screening of drugs was combined with a chemical genomics analysis of drug response signatures to identify the candidates with the highest inhibitory potential against the VCP/p97 ATPase.

First, we conducted a virtual screening of a total of ~2,900 withdrawn and FDA-approved drugs against the allosteric site of the VCP/p97 by molecular docking. The highest-scoring candidates were then screened for their potential ability to induce ER stress based on the gene expression signatures of their response derived from the connectivity map (CMAP)^30^ resource. Eleven drugs that displayed strong *in silico* binding to the VCP/p97 allosteric site and an ER stress signature in CMAP were then tested experimentally for their ability to inhibit the VCP/p97 ATPase activity *in vitro* and induce ERAD *in vivo*^31^. Importantly, our approach revealed three drugs, astemizole, clotrimazole and ebastine, as potential direct inhibitors of VCP/p97 and ERAD. Our findings strongly suggest the potential high efficiency (40% rate) of our integrated approach in the drug discovery and repositioning process.

## Results

### Structure based virtual screening

We started by mapping the allosteric binding site for NMS-873, a recently characterized compound with a high specificity and potency against VCP/p97, on the structure of the VCP/p97 protein (PDB ID: 3CF1^32^). According to the experimental data^6^, this site includes the residues Lys 615 and Asn 616, which are located at the middle of the interface of the VCP/p97 monomers. We employed a blind docking protocol (see Methods) to identify potential binding-sites on the VCP/p97 structure. The best poses on the more promising binding-sites were subsequently refined by running independent dockings on the selected binding sites. The blind docking protocol identified four sites around Lys 615 and Asn 616 for NMS-873. Then, we used a set of previously reported allosteric inhibitors for VCP^6^ to evaluate how well they are ranked at each site according to the experimental data. The results revealed that one site was able to rank the inhibitors properly according to the experimental data (Table 1). Therefore, the docking protocol we implemented to identify the sites targeted by these inhibitors was consistent with the previously reported experimental results^6^.

**Figure 1:**
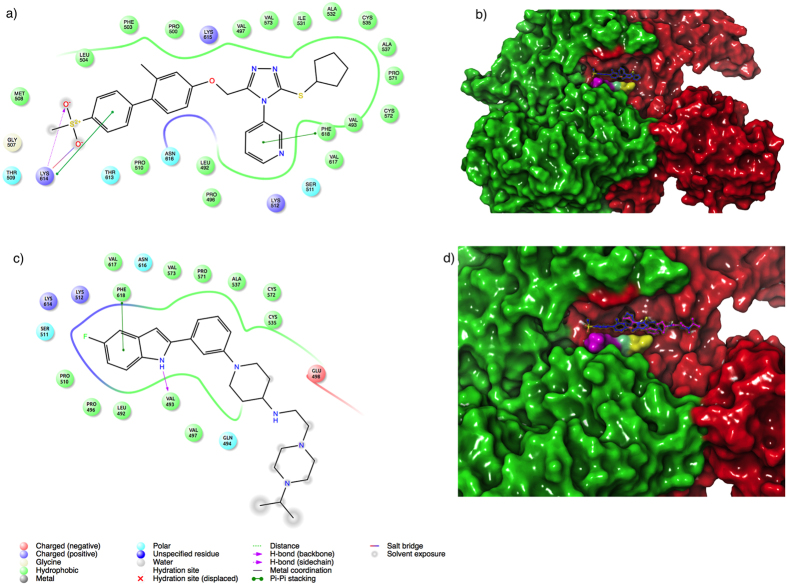
(**a**) 2D plot of VCP/p97 interactions with NMS-873 inhibitor and (**b**) its docked pose into the predicted binding site. (**c**) 2D plot of interactions between UPCDC30245 inhibitor and VCP/p97 and (**d**) the docked pose of NMS-873 and the experimental pose UPCDC30245 inhibitor (magenta) into the VCP/p97 binding site. Each monomer of VCP/p97 is in color green and red, respectively. The surface of Lys 614, Lys 615 and Asn 616, critical residues for the allosteric binding, is indicated in color magenta, cyan and yellow, respectively.

The interactions of NMS-873 and residues of the predicted binding-site are dominated by a mix of positively charged, polar and hydrophobic residues. In addition to Lys 615 and Asn 616, the analysis revealed a critical interaction with Lys 614 by hydrogen bonding and salt bridge, and a π-π stacking interaction with Phe 618 (Fig. 1a and b).

Thus, we performed molecular docking using the parameters of the identified site and 2,924 structures, including FDA-approved and withdrawn compounds, from the ZINC database^33^ as ligands.

The docking scores that we obtained with AutoDock 4.2^34^ were normalized and used to generate Z-score values for each ligand (see Supp. Table S1). Top 100 compounds were considered as potential binders of VCP/p97 and selected for further analysis.

During the preparation of this manuscript a high resolution Cryo-EM structure of VCP/p97-inhibitor complex was solved^7^ (PDB ID: 5FTJ). We decided to perform a comparative analysis of the NMS-873 binding-site predicted by us and the corresponding new inhibitor UPCDC30245. We applied the same blind docking protocol mentioned above by using the new VCP/p97 structure as the receptor and NMS-873 and UPCDC30245 as ligands. Such analysis enables us to verify where NMS-873 prefers to bind in the VCP/p97 structure and corroborate possible inconsistencies of our blind docking protocol. The results of this analysis showed that the RMSD value between the high-ranked predicted pose for the UPCDC30245 inhibitor and that observed in the Cryo-EM VCP/p97 structure was 1.73 Å, indicating high similarity between predicted and experimental binding pose. NMS-873 results were also consistent with our predicted binding site, as revealed by the RMSD values (0.96 Å) between predicted poses using the 3CF1 and 5FTJ structures, respectively. We found that our predicted binding sites for NMS-873 and the UPCDC30245 overlap each other in a region including the key residues for NMS-873 binding, Lys 614, Lys 615 and Asn 616, and probably indicating that it is critical for further development of new allosteric inhibitors (Fig. 1c and d). In spite of the compounds sharing their binding sites, they expand across in different directions. NMS-873 tends to orient preferentially towards the D1-D2 interdomain linker from one monomer, while UPCDC30245 orients mostly towards the region of the binding site that is formed by the adjacent monomers. These findings suggest that there is still room for the design of novel series of allosteric inhibitors that can bind in different regions of the allosteric binding site of VCP/p97.

### Chemical genomic analysis

We reasoned that the true VCP/p97 inhibitors among our top compounds obtained by virtual screening should also induce a cellular gene expression response consistent with the inhibition of ERAD and VCP/p97. To this end, we decided to leverage the collections of gene-expression data from the CMAP^30^, a catalog of gene-expression profiles collected from human cells treated with FDA-approved and withdrawn compounds as well as different well-characterized chemical reagents. Comparing the gene expression response profile of a known drug to those in this compendium has the potential to identify drugs with novel previously unknown functions. To identify drugs in the CMAP compendium with the potential to inhibit VCP/p97, we first leveraged the gene expression data obtained from the treatment of 293T cells with Eeyarestatin, a known inhibitor of VCP/p97^35^. Correlation of the gene expression response signature of Eeyarestatin to each of the 6100 genomic signature profiles in CMAP reveals drugs involved in proteasome inhibition (MG-132, MG-262, withaferin, celastrol) and ER stress induction (thapsigargin, geldanamycin) among the top drugs (see Supp. Table S2), confirming the validity of our approach and the power to detect potential inhibitors of ERAD. Importantly, the signatures of four drugs (niclosamide, ivermectin, clotrimazole, astemizole) in our list of potential VCP/p97 binding drugs from the docking analysis were among the top 50 drugs with the highest functional similarity to Eeyarestatin (Table 2). Two more drugs (iloprost and saquinavir), also displayed a statistically significant similarity to the Eeyarestatin profile (Table 2), though at a lower level of significance. These analyses suggest that these 6 drugs might be *bona fide* inhibitors of the VCP/p97 protein. However, the gene expression data from CMAP are not available for some of our high priority candidate drugs from the virtual screening, such as ebastine, which was ranked at the top 2 of our candidate list. Importantly though, ebastine belongs to the same class of compounds (H1 antihistamine) as astemizole, one of the six compounds whose genomic signature displayed a significant similarity to Eeyarestatin. Therefore, we decided to include ebastine in the further experiments because it possesses a very favorable safety profile in the market. Overall, the seven resultant compounds were selected for experimental validation.

### Experimental validation

First, the activities of these compounds against VCP/p97 in a cell-based degradation assay^31^ were evaluated. For this purpose, we used a dual-reporter system to monitor the ubiquitin-proteasome system (UPS) and VCP/p97 function in mammalian cells. This system comprises an ubiquitin fusion degradation (UFD) reporter Ub^G76V^-GFP and the oxygen-dependent degradation domain of HIF1α fused to luciferase (ODD-Luc), a non-ERAD substrate for proteasomal degradation. Measurement of GFP and luciferase activity in response to a treatment allows to assess the effect on p97-dependent and p97-independent proteolysis.

NMS-873 and MG132 were used as positive controls for p97-dependent and –independent protein degradation, respectively, and DMSO was used as a negative control. NMS-873 only displayed inhibition of the degradation of the Ub^G76V^-GFP reporter, while MG132, a proteasome inhibitor, inhibited degradation of both reporters with similar IC_50_ values. Importantly, ebastine, ivermectin, clotrimazole, saquinavir, and astemizole exhibited preferential inhibition of degradation of the p97 substrate Ub^G76V^-GFP (Table 3), although ebastine, astemizole and saquinavir also inhibited ODD degradation (Table 3). However, only clotrimazole and ivermectin displayed a strong selectivity for the inhibition of the p97 substrate, suggesting that these compounds may be specific direct inhibitors of VCP/p97.

In order to test direct inhibition of VCP/p97 ATPase activity by these compounds, we performed *in vitro* ATPase assays of the five UPS and VCP/p97 perturbing compounds to evaluate their inhibitory activity against VCP/p97. DBeQ and NMS-873, known VCP/p97 inhibitors, were used as positive controls. We found that three out of five compounds (60%) had direct activity against VCP/p97. Clotrimazole, an antifungal drug, was the compound with the highest potency against VCP/p97 ATPase (Table 4), and which also displayed the highest specificity for the p97-dependent substrate in the cell based assay.

**Figure 2:**
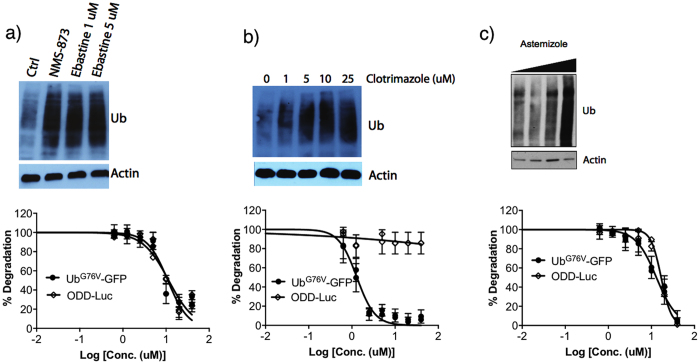
Western blot of polyubiquitinated proteins in MDA-MB-453 cells and, Ub^G76V^-GFP and ODD-Luc degradation in response to increasing concentrations of (**a**) ebastine, (**b**) clotrimazole and (**c**) astemizole, respectively. NMS-873 inhibitor was used as positive control.

However, ivermectin did not show activity against VCP/p97, suggesting that its mechanism of action (MOA) might be associated with the inhibition of the components of UPS other than VCP/p97 and the proteasome. Astemizole and ebastine, both H1 antihistamines, showed activity against VCP/p97 to varying degrees, suggesting that H1 antihistamine drugs might be explored to develop more potent VCP/p97 inhibitors. These results suggest that the list of compounds predicted by our computational approach is enriched for true hits. Importantly, these three compounds also induced the accumulation of polyubiquitinated proteins in cells, a hallmark of UPS inhibition, again consistent with their ability to inhibit UPS and VCP/p97 (Fig. 2).

### Mechanisms of VCP/p97 inhibition by the repositioned drugs

**Figure 3:**
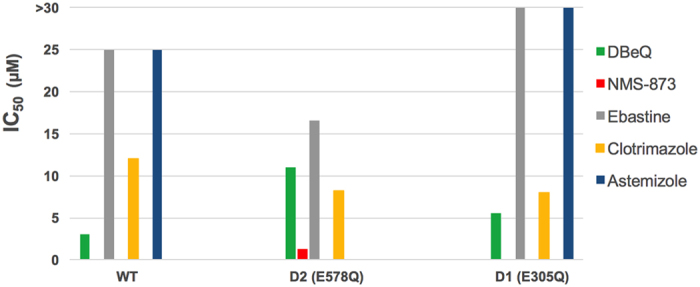
Inhibition of ATPase activity of WT VCP/p97 and the two D1 and D2 mutants by the three repositioned drugs in this study. DBeQ and NMS-873 were used as positive controls.

In order to explore the mechanisms of VCP/p97 inhibition by these compounds, we performed the *in vitro* ATPase assays by employing two Walker B (DExx box) motif mutants of the VCP/p97 protein: a D2-active mutant (E305Q) and a D1-active mutant (E578Q). Both mutants are able to assemble into their native structures similar to wild-type VCP/p97, but they lack the ATPase activity at the D1 or the D2 domain, respectively. The three compounds identified in this study as novel VCP/p97 inhibitors were screened for their potential to suppress the ATPase activity of the D2 and D1 active mutants. The IC_50_ values of DBeQ increased 3.7 and 1.8-fold against the D2, and the D1 mutants, relative to wild-type VCP/p97. NMS-873 showed the same inhibition for the D1 mutant, but inhibited D2 mutant with a 38-fold less potency than the WT VCP/p97 (Fig. 3). These results for DBeQ and NMS-873 are consistent with the previous results showing that the former inhibits both D1 and D2 ATPase activities, while NMS-873 was sensitive to the D2 mutation, but was insensitive to D1 mutations^36^. The IC_50_ values for the two domain mutants for astemizole and ebastine suggest that they affect the ATPase activity of VCP/p97 with different mechanisms. Astemizole’s ATPase-inhibitory activity decreased in both D1 and D2 mutants and ebastine showed more activity toward D1-active mutant (E578Q) but no activity toward D2-active mutant (E305Q) (Fig. 3). However, clotrimazole inhibited both mutants with a similar potency as for WT VCP/p97, suggesting that clotrimazole inhibited the catalytic activities of both domains (Fig. 3).

**Figure 4:**
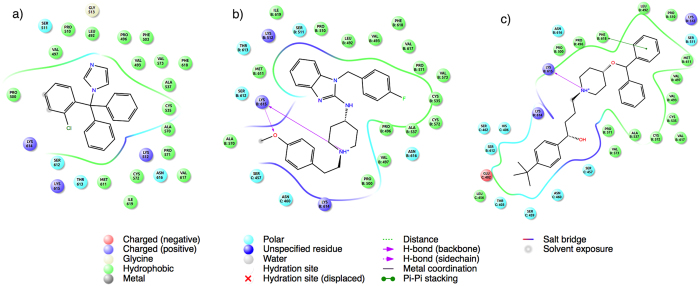
2D plots of VCP/p97 interactions with three repositioned drugs found in this study. (**a**) clotrimazole, (**b**) astemizole, and (**c**) ebastine.

To further characterize the binding modes of the three repositioned drugs, we performed a detailed analysis of their docked poses on the binding site of the VCP/p97 protein. Interestingly, clotrimazole and astemizole mostly bind to one VCP/p97 monomer in a similar manner to NMS-873, but ebastine instead seemed to bind at the interface of two monomers, suggesting that the compound might perturb the VCP/p97 multimer complex formation. The interactions of the three compounds and VCP/p97 protein indicated that they are mediated, first, by the interactions with the positive and hydrogen donor/acceptor hot spot formed by Lys 614 and 615 residues on VCP/p97, and second, by interactions with a set of polar and hydrophobic residues (Fig. 4a–c).

## Discussion

VCP/p97 is an attractive therapeutic target for cancer and neurodegenerative diseases. Thus, potent and specific inhibitors of VCP/p97 have been identified by high-throughput screening (HTS) and chemical optimization^6^,^8^,^9^. Typically, the VCP/p97 inhibitors fall into two classes: ATP-competitive inhibitors and non-competitive inhibitors. Despite advances in the development of VCP/p97 inhibitors, there are currently no clinically available drugs in the market targeting the VCP/p97 or any of the ERAD components. Here, by employing an integrative computational approach followed by experimental validation, we have identified drugs that are able to perturb the UPS, thereby repositioning these three drugs as VCP/p97 inhibitors. These drugs include two H1 antihistamine (astemizole and ebastine) and one antifungal drug (clotrimazole).

The cell-based assay allows us to stratify the compounds by their ability to inhibit VCP/p97-dependent and VCP/p97-independent proteasome substrates. Astemizole and ebastine inhibited both p97-dependent and independent substrates, highlighting their potential to inhibit at least one component of the UPS in addition to VCP/p97. This is confirmed by ATPase assays were both drugs showed inhibitory activity against VCP/p97 at IC50 < 25 μM. Conversely, clotrimazole displayed specificity against VCP/p97-dependent proteasome substrates (IC50 < 1.4 μM) and inhibition of ATPase activity (IC50 < 12 μM) that was comparable to the known VCP/p97 inhibitors (DBeq and NMS-873).

Recent reports have shown that VCP/p97 is a dynamic protein consisting of two ATPase domains, D1 and D2, which work together to drive the processing of protein substrates^36^,^37^,^38^,^39^,^40^. Such reports revealed that these domains communicate with each other to critically control the capacity of both domains to hydrolyze ATP.

In this study, we provide an analysis of the selectivity and the mechanism of inhibition of the three repositioned drugs for the D1 and D2 ATPase domains of VCP/p97, by using active mutants with point mutations at the conserved Walker A and B positions of D1 and D2, respectively. Our results showed that these three drugs exhibit different selectivity against the VCP/p97 active mutants, suggesting a different mechanism of inhibition.

Astemizole showed an inhibition profile similar to NMS-873, more sensitive to D2 mutations, but it was still sensitive to changes in D1^36^. For example, the D1 mutation increased the IC50 for astemizole by ~2-fold, suggesting that the architecture of the D2 active site was changed in response to the mutation and/or by the nucleotide binding to D1, and probably that astemizole prefers binding to VCP/p97 in the absence of the nucleotide in both domains. Ebastine was sensitive to D1 mutations, suggesting that it is mainly targeting this domain. It is worth noting that D1 ATPase activity alone is difficult to block as showed elsewhere^36^. Interestingly, the IC50 of ebastine decreased in response to the D2 mutation by ~2-fold, suggesting that the nucleotide binding at D2 domain induce a conformational change in the structure in VCP/p97 and increasing the affinity of ebastine against it. These results are consistent with previous findings about the cross-inhibitory effect on the activity of the D1 and D2 domains^36^.

Unexpectedly, clotrimazole displayed an improvement in the IC50 (from 12.0 μM to 8.0 μM) for both mutants, suggesting that clotrimazole has higher affinity for VCP/p97-substrate bound state, freezing VCP/p97 into a conformation that may have also prevented ATP/ADP release from both domains. This observation is consistent with the inhibition mechanism against VCP/p97 proposed for NMS-873 and UPCDC30245 allosteric inhibitors^6^,^7^.

By considering the differences observed in the IC50 between the cell-based assays and the ATPase inhibition assay (i.e. lower IC50 *in vivo* vs *in vitro*), our results suggest that these drugs mediate their effects through binding different families of on- and off-target effectors, rather than solely through VCP/p97. For example, the oral administration in mice of NMS-873, a specific and potent VCP/p97 inhibitor, showed modest systemic exposure and modest bioavailability, suggesting issues related to extensive first pass metabolism and/or solubility^41^. Thus, *in vivo* efficacy is not necessarily correlated with specific and high binding affinity as suggested previously^42^.

Several reports have suggested anti-cancer activities of the three repositioned drugs. For example, astemizole has shown anti-cancer activity for cervical cancer, hepatocellular carcinoma, melanoma and breast cancer^43^,^44^,^45^,^46^. These reports suggested that the mechanism associated with the anti-cancer activities of astemizole were due to its on-target (H1 receptor) and off-targets (Erg potassium channels), as the histamine receptors and potassium channels have been found to play important roles in tumor cell homeostasis^47^,^48^,^49^,^50^. More recently, Ellegard *et al*.^51^, have conducted a screening of a cationic amphiphilic drug (CAD) library for cytotoxicity against non-small cell lung cancer (NSCLC) cells and identified several CAD antihistamines as inducers of lysosomal cell death. They found that sub-micromolar concentrations of astemizole and ebastine sensitized NSCLC cells to chemotherapy and reverted multidrug resistance in NSCLC, breast and prostate cancer cells.

Several reports have indicated that clotrimazole has anti-cancer properties by decreasing hexokinase (HK) binding to the outer mitochondrial membrane, detaching phosphofructokinase-1 (PFK-1) and aldolase from the cytoskeleton, and by interfering with Ca2+ metabolism and blockage of the Ca2+ activated potassium channel^52^,^53^,^54^,^55^,^56^. Motawi *et al*.^57^, revealed that the combination of imatinib (an anti-cancer drug) and clotrimazole enhances cell growth inhibition in breast cancer cell lines.

Although there is not much information about the anti-cancer properties of ebastine, we suggest that its potential as an anti-neoplastic agent should be explored further due to its potential ERAD-inhibitory function and a remarkable safety/tolerability profile in patients.

Astemizole has been withdrawn from the market because of rare cases of cardiovascular side effects^58^ and clotrimazole exhibits poor water solubility that reduces its bioavailability. Nevertheless, both drugs stand as molecules with high potential to be repositioned as anti-cancer drugs.

For example, different efforts have been performed in order to improve solubility of clotrimazole by using of microcapsules, liposomes, nanospheres, suspensions with hydroxypropylmethyl cellulose and more recently, by using beta-cyclodextrins showed an enhanced solubility and a greater bioavailability of clotrimazole in rats^59^,^60^,^61^. Most of the astemizole toxicity cases involved overdosing of above 200 mg per day, which is far above the recommended dose of 10 mg once daily^49^. Downie *et al*.^49^, demonstrated that a dose of 50 mg/kg is sufficient to inhibit tumor growth and was well tolerated in mice without producing evident side effects. Clinical trials should be performed to determine how well astemizole and clotrimazole are tolerated by cancer patients and their real potential as repositioned anti-cancer drugs.

We considered that the future development of clotrimazole and astemizole derivatives should center on retaining their anti-cancer activities while increasing bioavailability and safety, respectively, by the rational integration of information from structure relationship studies, biochemical/cell based assays and patient medical records.

The results presented in this study contribute to the molecular understanding of the polypharmacological profile exhibited by the three drugs repositioned in this study and suggest that their anti-cancer activities are a consequence of complex interactions between multiple intracellular components. Therefore, they might be considered as multi-target agents, whose pharmacological response is associated to the modulation of multiple targets/pathways, which may be advantageous against complex diseases like cancer.

## Conclusions

We have developed a holistic approach including *in silico* integration of structure-based virtual screening and chemical genomics data from CMAP to reposition drugs as novel inhibitors of VCP/p97 receptor. Testing these new compounds in a series of cell-based and biochemical assays demonstrated inhibition against VCP/p97; activity not previously described for these drugs.

Our repositioned compounds represent an opportunity to speed up the drug discovery process for therapeutic intervention in neurodegenerative diseases and cancer which importantly, will take significantly less time to translate into the clinic. In addition, they would be relevant probe molecules to study the function of the UPS and ERAD, and starting points for future optimization into new derivatives with improved safety profile and efficacy.

## Methods

### Docking

The structure of VCP/p97 was obtained from Protein Data Bank (PDB ID: 3CF1^32^). Auto-DockTools^34^ was used to add the Gasteiger charges and polar hydrogens to VCP/p97.

For the blind docking, we adapted the protocol reported by Hetényi and vander Spoel^62^,^63^.

All the docking simulations were performed Autodock 4.2 suite^34^. A grid box with the center close to side chain of the residue Asn 616, critical residue for the binding of NMS-873 inhibitor, was set to 80 grid points in each dimension (x, y and z) with 0.55-Å and the grid maps were obtained.

The tridimensional structure of the NMS-873 inhibitor was built with Avogadro software^64^. We obtained the conformation associated with the lowest energy by conjugated gradient minimization using the same software. Once the structure was energetically stable, we prepared it (add hydrogens and charges) with UCSF Chimera software^65^. Auto-DockTools was used to obtain the pdbqt file of NMS-873 inhibitor.

The docking parameters for the blind docking were: population size of 250, 20 million energy evaluations and 200 runs. The other Autodock default optimization parameters were maintained for docking simulation. The best sites according to the poses with lowest energy were selected to perform focused docking simulations. For such simulations, we reduced the grid points to 48, 68 and 52, in x, y and z, respectively, with 0.375-Å of spacing. We modified the above docking parameters as follows: population size of 150, 5 million energy evaluations and 100 runs. The best site for NMS-873 binding according to the pose with lowest energy and number of clustered conformations was the one whose center is located in 100.137, 2.863 and 36.157 (x, y and z, respectively). This site and its associated parameters were used for the virtual screening by docking simulations.

A library of 2,924 structures of compounds, including approved and withdrawn drugs, were retrieved from ZINC database^33^. Docking was performed into the predicted binding site for NMS-873. AutoDockTools was used to generate pdbqt files for the compounds in the library, hydrogens and charges were kept from original files of the ZINC database. The scoring results of AutoDock were normalized by calculating the Z-score as follows


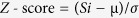


where *Si* is the scoring value of a Autodock scoring function, μ is the mean value, and σ is the standard deviation of the scoring function. The top 100 compounds were selected for chemical genomics analysis (Z-scores < −2).

### Chemical genomics analysis

Pre-processed data matrices were obtained from CMAP^30^ (http://www.broadinstitute.org/cmap/). To generate a signature of VCP/p97 inhibition, we leveraged the microarray gene expression dataset of 293 T cells treated with Eeyarestatin^35^. Our signature comprised genes with log ratio values of expression change greater than |0.5|, which amounted to 1758 genes. To score each CMAP instance for similarity to this signature, we carried out Pearson correlation of the CMAP expression change values with the log ratio values of the 1758 genes. The results for all the instances are given in the Supplementary Table S2. Then, we matched the drugs with significant results against our list of potential inhibitors of VCP/p97 obtained by docking and were selected for the experimental validation.

### Ub^G76V^-GFP and ODD-Luc degradation assays

A dual-reporter stable HeLa cell line was generated to monitor UPS function in mammalian cells^31^. A ubiquitin fusion degradation (UFD) reporter Ub^G76V^-GFP and an oxygen-dependent degradation domain of HIF1α fused to luciferase (ODD-Luc). We could identify molecules that selectively block p97 activity but no other general components of the UPS by seeking compounds that stabilize Ub^G76V^-GFP but not ODD-Luc. Cells were seeded on 384-well plates (3000 cells/well) and grown for 18 h. Cells were treated with modified DMEM (without phenol red, folic acid, riboflavin, and vitamin B12) containing MG132 (4 μM) for 1 h and washed twice with pre-warmed PBS. Modified DMEM containing FBS (2.5%), CHX (50 μM), and DMSO or a test compound (0, 0.625, 1.25, 2.5, 5, 10, 20, 40 μM) was added to each well. Plates were imaged on the ImageXpress Micro microscope (Molecular Devices) after 160 min. Luciferase activity were measured by adding D-Luciferin (10 μl of 2.5 mg/mL in PBS) into each well containing 38 μl of medium and incubated at room temperature for 10 min. Use Synergy Neo (BioTek) to read Luciferase signal.

## Additional Information

**How to cite this article**: Segura-Cabrera, A. *et al*. A structure- and chemical genomics-based approach for repositioning of drugs against VCP/p97 ATPase. *Sci. Rep.* **7**, 44912; doi: 10.1038/srep44912 (2017).

**Publisher's note:** Springer Nature remains neutral with regard to jurisdictional claims in published maps and institutional affiliations.

## Supplementary Material

Supplementary Tables

## Figures and Tables

**Table 1:** Docking results for the predicted binding site of the allosteric inhibitors.

Inhibitor	Autodock 4 score (kcal/mol)	Autodock 4 *Ki* (μM)	Experimental IC50 (μM)^6^
NMS-873	−9.83	0.06	0.03
NMS-249	−8.56	0.54	0.29
NMS-862	−6.47	18	2.66

**Table 2:** Compounds predicted by docking as VCP/p97 inhibitors and significant similarity to the Eeyarestatin signature.

Compound ID	Compound Name	Docking Z-score	Correlation with Eeyarestatin signature (Pearson’s r)	*P*-value of correlation
ZINC03781952	Ebastine	−6.92	NA	NA
ZINC26664090	Saquinavir	−6.29	0.11	1.38E-06
ZINC00601274	Astemizole	−5.98	0.3	8.40E-39
ZINC64858024	Iloprost	−4.11	0.05	0.02
ZINC03874496	Niclosamide	−3.37	0.387	5.79E-64
ZINC03807804	Clotrimazole	−2.77	0.33	4.02E-46
ZINC245224134	Ivermectin	−2.12	0.37	7.70E-59

**Table 3:** IC_50_ values (μM) of cell-based assays for the compounds predicted by docking and CMAP analysis.

Compound	UbG^76V^-GFP IC_50_	ODD-Luc IC_50_
MG132^a^	1.2	4.4
NMS-873^a^	1.4	>40
Niclosamide	>40	>40
Ivermectin	9.7	>40
Clotrimazole	1.4	>40
Saquinavir	10.7	20.8
Astemizole	13.1	16.9
Iloprost	>40	>40
Ebastine	12.0	12.6

^a^Positive control.

**Table 4:** IC_50_ values (μM) for the selected compounds against WT, E305Q, or E578Q VCP/p97 ATPase activities at 200 μM ATP obtained with 8-dose titration.

Compound	WT	D2- Walker B mutant	D1- Walker B mutant
		E578Q	E305Q
DMSO	>30	>30	>30
DBeQ^a^	3	11.0	5.5
NMS-873^a^	0.038	1.3	0.038
Ebastine	25	16.6	>30
Ivermectin	>30	>30	>30
Clotrimazol	12	8.2	8.0
Saquinavir	>30	>30	>30
Astemizole	25	>30	>30

^a^Positive control.
